# Border Disease Virus among Chamois, Spain 

**DOI:** 10.3201/eid1503.081155

**Published:** 2009-03

**Authors:** Ignasi Marco, Rosa Rosell, Oscar Cabezón, Gregorio Mentaberre, Encarna Casas, Roser Velarde, Santiago Lavín

**Affiliations:** Universitat Autònoma de Barcelona, Bellaterra, Spain (I. Marco, O. Cabezón, G. Mentaberre, E. Casas, R. Velarde, S. Lavín); Centre de Recerca et Sanitat Animal and Generalitat de Catalunya, Barcelona, Spain (R. Rosell)

**Keywords:** Chamois, Rupicapra pyrenaica pyrenaica, border disease virus, pestivirus, mortality, Spain, dispatch

## Abstract

Approximately 3,000 Pyrenean chamois (*Rupicapra pyrenaica pyrenaica*) died in northeastern Spain during 2005–2007. Border disease virus infection was identified by reverse transcription–PCR and sequencing analysis. These results implicate this virus as the primary cause of death, similar to findings in the previous epizootic in 2001.

Chamois (genus *Rupicapra*) are goat-like bovids native to the mountain areas of Europe and the Near East; they have also been introduced into New Zealand. In 2001 and 2002 a new pestivirus (family *Flaviviridae*) was associated with an outbreak of a previously unreported disease in Pyrenean chamois (*R*. *pyrenaica pyrenaica*) at the Alt Pallars-Aran National Hunting Reserve in the Pyrenees in northeastern Spain (Figure 1) (*1*). Molecular characterization assigned this virus to the border disease virus (BDV) cluster, 1 of the 4 main species of the genus *Pestivirus* (*2*,*3*). Later studies showed that the disease has become endemic in the area and could have a serious effect on chamois population dynamics (*4*,*5*).

**Figure 1:**
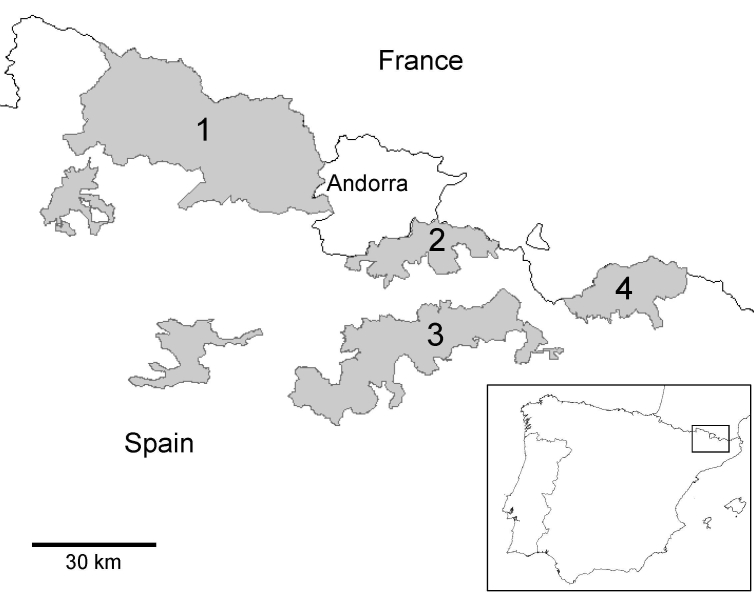
Map of northeastern Spain showing the National Hunting Reserves in Catalonia (shaded areas): 1, Pallars-Aran; 2, Cerdanya-Alt Urgell; 3, Cadí; 4, Freser-Setcases.

## The Study

In December 2004, an adult male chamois was found alive with respiratory disease at the Cerdanya-Alt Urgell National Hunting Reserve, ≈30 km southeast of a previously affected area (Figure 1). At the beginning of 2005, sudden deaths of chamois were observed in the same area. Dozens of carcasses were found in February and March. Three animals captured alive showed severe clinical signs of respiratory disease. Later, 2 isolated sick chamois found in May and October had mainly clinical signs of cachexia and alopecia, similar to those previously observed in 2001 and 2002 (*1*).

A census conducted in July 2005 corroborated the collapse of the chamois population, which decreased from 563 chamois in 2004 (preoutbreak) to only 81 chamois. Thus, the estimated cumulative rate of decrease in this area would have been 85.6%. In June 2005, the disease spread to the nearby Cadí National Hunting Reserve and private hunting areas (Figure 1), triggering another episode of mass deaths that has lasted for ≈31 months. An area >125,000 hectares of chamois habitat was affected, with the population decreasing from 3,458 chamois in 2004 (preoutbreak) to 1,281 in July 2006 (estimated cumulative rate of decrease 63%).

We studied 68 affected chamois (41 males and 27 females, age range 1–15 years), 6 from Cerdanya-Alt Urgell and 62 from Cadí Reserve. The main clinical signs and lesions of sick animals were cachexia, alopecia, and respiratory disease (Table 1). We performed necropsies on all 68 chamois. Apart from cachexia in most animals, bronchopneumonia was the main macroscopic lesion (in 31 chamois); 4 had diarrhea, 2 had infectious keratocojunctivitis, 2 had an abscess, and 1 had severe fibrinous pleuropericarditis.

**Table 1:** Characteristics of 23 ill Pyrenean chamois, Spain, 2005–2007*

Chamois no.	Date	Clinical symptoms	ELISA result for pestivirus	RT-PCR result for pestivirus	Isolate and origin	GenBank accession no.
1	2004 Dec	Pneumonia	–	+	Cerdanya-1	AM905930†
2	2005 Mar	Pneumonia	–	+	Cerdanya-2	AM905931
3	2005 Mar	Pneumonia	–	+	Cerdanya-5	NA
4	2005 Mar	Pneumonia	–	+	Cerdanya-3	AM905932†
5	2005 May	Cachexia, alopecia	–	+	Cerdanya-4	AM905933†
6	2005 Aug	Cachexia, pneumonia	–	+	Cadi-3	AM905920†
7	2005 Aug	Diarrhea, pneumonia	+	+	Cadi-13	NA
8	2005 Sep	Pneumonia, predation	+	+	Cadi-14	NA
9	2005 Sep	Predation	–	–	Cadi-15	NA
10	2005 Oct	Cachexia	–	+	Cadi-2	AM905919†
11	2005 Oct	Trauma	NA	–	Cadi-16	NA
12	2005 Oct	Cachexia, alopecia	–	+	Cerdanya-6	NA
13	2005 Dec	Cachexia, diarrhea	–	+	Cadi-4	AM905921†
14	2006 Feb	Cachexia, alopecia	–	+	Cadi-7	AM905924
15	2006 Feb	Cachexia, alopecia	–	+	Cadi-5	AM905922†
16	2006 Apr	Cachexia, alopecia	–	+	Cadi-10	AM905927
17	2006 Apr	Cachexia	–	+	Cadi-1	AM905918
18	2006 Apr	Cachexia, alopecia	–	+	Cadi-8	AM905925
19	2006 May	Cachexia, alopecia, pneumonia	–	+	Cadi-9	AM905926
20	2006 Jun	Cachexia, alopecia, pneumonia	–	+	Cadi-6	AM905923†
21	2006 Aug	Cachexia, alopecia, pneumonia	–	+	Cadi-11	AM905928
22	2006 Aug	Cachexia, alopecia, pneumonia	–	+	Cadi-12	AM905929
23	2007 Dec	Cachexia, pneumonia	–	+	Cadi-17	NA

*RT-PCR, reverse transcription–PCR; NA, not available. †Virus sequenced in this study.

Spleen and kidney homogenates were examined for pestivirus nucleic acid by reverse transcription PCR by using panpestivirus primers (Pesti 3 and Pesti D) (*6*,*7*). All chamois, with the exception of 2 animals from the Cadí Reserve, were positive for pestivirus.

**Figure 2:**
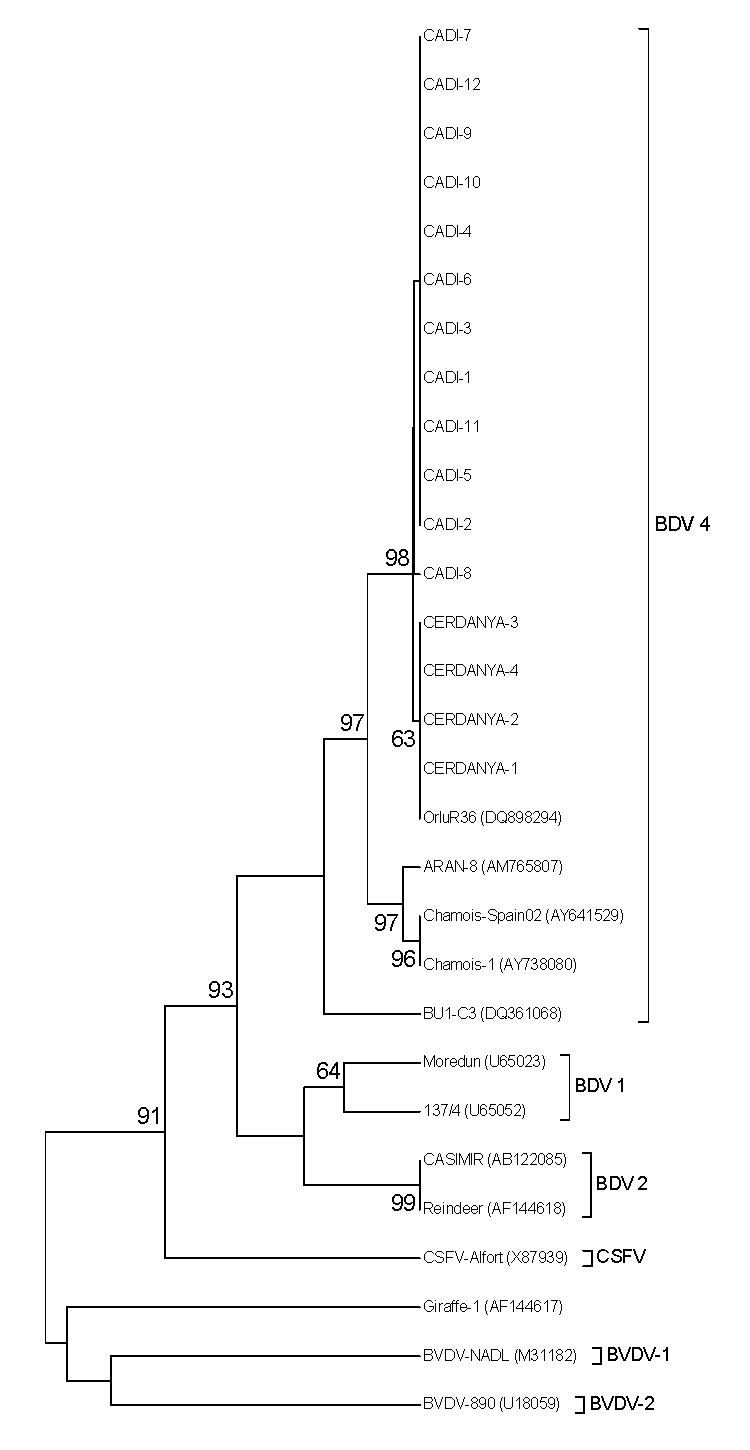
Unrooted neighbor-joining phylogenetic tree based on the 5′ untranslated region sequence among pestiviruses isolated from chamois, Spain. Chamois strains were enclosed in a differentiated group into border disease virus 4 (BDV-4). Numbers on the branches indicate percentage bootstrap values of 1,000 replicates. Numbers on the right in parentheses indicate GenBank accession numbers. CSFV, classical swine fever virus; BVDV, bovine viral diarrhea virus.

Sequence analyses of the 243-bp fragment of the 5′ untranslated region of 9 isolates were performed for phylogenetic analysis by using primers 324 and 326 (*8*). Purified amplicons (Minelute Gel Extraction Kit; QIAGEN, Hilden, Germany) were analyzed with the BigDye Terminator kit version 3.1 and the ABI 3130xl Genetic Analyzer (Applied Biosystems, Warrington, UK). Seven isolates had been sequenced in an investigation of BDV shedding and detection in organs of naturally infected Pyrenean chamois. The phylogenetic tree was constructed by the neighbor-joining method (*9*) by using automatic root location. Bootstrap analysis of 1,000 replicates was performed by creating series of bootstrap samples to test tree branch reliability. Resulting sequences showed that chamois were infected with the BDV-4 genotype and that isolates from the 2 outbreaks formed a discrete cluster separated from isolates of the previous outbreak (Figure 2).

Serum samples from 60 chamois were tested for pestivirus-specific antibodies by using an ELISA (Synbiotics, Lyon, France) that detects antibodies against a protein (p80/125) common to all bovine viral diarrhea virus (BVDV) and BDV strains. Only 2 PCR-positive chamois had antibodies, which suggests that most of them could have been persistently infected. In this condition, animals become infected during early pregnancy, are immunotolerant and seronegative, and release large amounts of virus into the environment, thus being the main source of transmission. Retrospectively, serum samples of 78 healthy chamois captured at the Cadí Reserve during 2000–2002 were also tested and showed positive results in 4 (5.1%) chamois.

To confirm ELISA results and determine antibody specificity, serum samples from 6 ELISA-positive chamois were tested by using a comparative virus neutralization test. Viral strains tested were BVDV-1 strain NADL, BVDV-2 strain atypical, BDV strain Spain 97 (*10*), BDV strain Moredun, BDV strain 137/4, and BDV strain CADI-6 (chamois). Neutralizing antibody titers were expressed as the reciprocal of the highest dilution that neutralized 100 tissue culture infective doses in all cultures. Titers >10 were considered positive. Viral replication was monitored by immunoperoxidase monolayer assay with a polyclonal pestivirus-specific antibody. The comparative virus neutralization test confirmed ELISA results in all 6 chamois. Higher titers to BDV Cadi-6 were observed in the 2 pestivirus RT-PCR–positive chamois. Higher titers to BDV Spain 97 were also observed in most healthy animals, which suggest infection with strains of ovine origin (Table 2).

**Table 2:** Virus neutralization titers of against 6 pestivirus strains in serum samples from 6 chamois, Spain, 2005–2007*

Chamois no.	BDV Spain 97	BDV Cadi-6	BDV 137/4	BDV Moredun	BVDV-1 NADL	BVDV-2 atypical
1 (PCR positive)	20	160	0	0	0	0
2 (PCR positive)	0	160	0	0	0	0
3 (healthy)	320	160	160	40	40	0
4 (healthy)	320	80	80	40	40	0
5 (healthy)	0	0	0	40	0	10
6 (healthy)	80	80	40	0	0	0

*BDV, border disease virus; BVDV, bovine viral diarrhea virus.

## Conclusions

BDV infection in chamois in the Cerdanya-Alt Urgell Reserve could have been the result of the spread of the disease reported in the Alt Pallars-Aran Reserve in 2001 and 2002. The extreme severity of the disease in the Cerdanya-Alt Urgell and Cadí Reserves is unprecedented in pestivirus infections in wild ruminants (*11*). Clinical and pathologic findings suggest that pneumonia was a major contributing factor for the high mortality rate observed at the Cerdanya-Alt Urgell Reserve. Bronchopneumonia is frequently found in chamois (*12*), but this problem may have been magnified by immunosuppressive effects of coincident BDV infection. The severity of the outbreak may have been affected by lack of immunity at the population level. Data from the Cadí Reserve before the outbreak showed low antibody seroprevalence. In comparison, in the Freser-Setcases National Hunting Reserve, ≈8 km from Cadí, seroprevalence was 71% in 2003. In this reserve, the same virus was identified in a healthy chamois in September 2006 and in an isolated diseased chamois in June 2007. However, to date no epidemics have been reported.

After these 2 outbreaks in Cerdanya-Alt Urgell and Cadí Reserves, the remaining population may have acquired immunity against the infection, as was the case after the first outbreak in the Alt Pallars-Aran National Hunting Reserve (*4*). The recovery rate in the Cerdanya-Alt Urgell and Cadí chamois populations has not been as fast as expected. In July 2007, the census in these 2 populations identified 153 and 1,616 chamois, respectively. In July 2008, only 165 and 1,661 chamois, respectively, were identified.

These results implicate BDV infection as the primary cause of death in chamois, as previously reported in 2001 and 2002. Further experimental studies are ongoing to fulfill criteria needed for a specific microorganism to be identified as the cause of this disease. Additional studies are needed to determine whether this infection will have a negative effect on the population dynamics of Pyrenean chamois and epidemiologic relationships between chamois and sheep with respect to infection with different BDV strains.
